# Parental Involvement and Life Satisfaction in Early Adolescence

**DOI:** 10.3389/fpsyg.2021.628720

**Published:** 2021-02-17

**Authors:** Mauricio Salgado, Luis González, Alejandra Yáñez

**Affiliations:** ^1^Centre for Research in Inclusive Education, School of Social Sciences, Universidad Andres Bello, Santiago, Chile; ^2^School of Psychology, Pontificia Universidad Católica de Valparaíso, Valparaíso, Chile

**Keywords:** early adolescence, Chile, gender, parental involvement, life satisfaction

## Abstract

Early adolescence is a developmental stage that comprises some basic interactional processes with parents, which can be described as gaining autonomy while maintaining relatedness. Studying how maternal and paternal involvement influence the life satisfaction of sons and daughters during early adolescence is especially important while seeking to understand the challenges of this developmental stage. In this paper, we investigate the differential effects of maternal and paternal involvement, as assessed by sons and daughters, on their life satisfaction during early adolescence. We use a unique survey conducted in Chile, *The National Survey on Student Trajectories and Transitions*, focusing on a subsample of 497 early adolescents attending 5th to 8th grade (60% female, *M* age = 12.42, SD = 1.18). Our findings indicate that both paternal and maternal involvement are positively correlated with the life satisfaction of adolescents. We also find that the gender of adolescents moderates the effect of maternal involvement, so daughters (but not sons) who deemed the involvement of their mothers to be more positive reported greater life satisfaction. More positive paternal involvement correlates with greater life satisfaction for sons and daughters. We discuss some mechanisms that might bring about these differences.

## Introduction

Research on parental involvement has found that adolescents with strong bonds with their parents tend to show better personal outcomes, such as academic performance, emotional development, and fewer behavioral problems (Senechal and LeFevre, 2002; Flouri and Buchanan, 2004; Day and Padilla-Walker, 2009; Benner et al., 2016; Kalil and Mayer, 2016). In early adolescence (10–14), the relationship with parents starts changing remarkably. The Individuation-Separation theory (Reis and Buhl, 2008) states that early adolescence is a developmental stage that comprises some basic interactional processes with parents, which can be described as gaining autonomy or separateness (i.e., independence from parental authority, the construction of a self that is detached from parental influence, and a change from unilateral authority to cooperation) while maintaining relatedness (i.e., respect for the parents, self-disclosure, a sense of obligation to the family, and a feeling of attachment to the parents). Thus, although early adolescents begin to spend increasingly more time with peers, they still need a close and supportive relationship with their parents (Galambos et al., 2003; Leung et al., 2004; Schwarz et al., 2012). Parents usually devote less time to interacting directly with them and more time to planning and monitoring their academic and social networks (Kalil et al., 2012). Indeed, expert recommendations, public policies, and scientific research underscore the importance of both paternal and maternal involvement on the well-being of adolescents (Young et al., 1995; Leung et al., 2004; Lamb, 2010; Schwarz et al., 2012; McLanahan et al., 2013). *Life satisfaction* is part of the multidimensional construct of subjective well-being. Subjective well-being refers to the self-evaluation of individuals and comprises emotional components such as positive affect and lack of negative affect, with life satisfaction as a cognitive component. Life satisfaction, in turn, encompasses global, and domain-specific judgements about the personal lives of individuals, such as friends, neighborhoods, schools, and family (Diener et al., 1985; Diener and Suh, 1997; Diener, 2006). There are already well-validated measures of life satisfaction in children and adolescents (e.g., Seligson et al., 2003; Alfaro Inzunza et al., 2015). Some studies using these measures support the association between parental involvement and life satisfaction of adolescents (Casas et al., 2007, 2012). These studies also suggest that the association is stronger in early adolescence compared to middle and late adolescence (Suldo and Huebner, 2004). Besides, in the last decade, researchers have been moving away from the general idea of parenting, toward mothering, and fathering, which are impacted both by the gender of the child, and the outcome being examined (Day and Padilla-Walker, 2009). However, most previous studies do not distinguish between maternal and paternal involvement, and usually overlook the potential moderating effect of the gender of the adolescents. Therefore, we still know little about the differential effect that maternal and paternal involvement might have on life satisfaction in early adolescence, and whether the gender of the adolescents provides the boundary conditions for the relationship between parental involvement and life satisfaction. In this paper, we aim to fill this gap in the literature using survey data from a sample of early adolescents who reported the (perceived) involvement of their mothers and fathers, and their life satisfaction. Studying how maternal and paternal involvement influence the life satisfaction of sons and daughters during early adolescence is especially important while seeking to understand the complexities and challenges of this developmental stage.

*Parental involvement* refers to a suit of behaviors which involve the time dedicated by mothers and fathers to meaningful and ritualistic activities, so children may feel secure, develop positive emotions, and increase their life satisfaction (Rask et al., 2003; Flouri and Buchanan, 2004; Levin and Currie, 2010). The emotions and judgments of adolescents, such as feeling close to their parents, spending enough time with them, and being aware that their parents are interested in their side of the story, are all important indicators of the perceived level and quality of maternal and paternal involvement. We aim at investigating the relationship between maternal and paternal involvement and the satisfaction of early adolescents with their lives, as measured by the *Brief Multidimensional Students' Life Satisfaction Scale* (BMSLSS, Seligson et al., 2003). Since it has been documented that there are gender differences in parent-children relations (Starrels, 1994; Raley and Bianchi, 2006), we take advantage of a unique Chilean survey—*The National Survey on Student Trajectories and Transitions*– which applied the BMSLSS to a sample of early adolescents who were also asked to judge maternal and paternal involvement, in order to be able to disentangle the effects of both measures on the self-reported life satisfaction of sons and daughters. We seek to examine two specific questions. First, are perceived paternal and maternal involvement correlated with life satisfaction in early adolescence? Second, is the potential relationship of maternal and paternal involvement with life satisfaction conditional to the gender of the adolescent? Two main hypotheses directed this study. Firstly, we predict that both maternal and paternal involvement have a positive correlation with the life satisfaction of early adolescents. Secondly, we predict that the child's gender provides the boundary condition for the relationship between parental involvement and the life satisfaction of adolescents. The rest of the paper is structured as follows: we review the literature on parental involvement using subjective well-being in general, and life satisfaction in particular, during late childhood and early adolescence, justifying our main predictions (section Parental Involvement and the Life Satisfaction of Early Adolescents). Then, we present the materials and methods used in this study, thoroughly describing the main measures, control variables, and the analytical strategy applied here (section Materials and Methods). Data analyses and main results are described later (section Results). Finally, we discuss the results and provide some concluding remarks (section Discussion and Concluding Remarks).

## Parental Involvement and the Life Satisfaction of Early Adolescents

Parental involvement is the act of taking part in activities or situations related with one's biological or putative children (Georgiou, 1996; Goodall and Montgomery, 2014; Salgado, 2015). The metric for parental involvement is usually time, but it is important to distinguish the child-related activities in which parents are involved (Kalil et al., 2012). Fathers are, on average, less involved than mothers, but devote more time to playing and teaching, whereas mothers perform significantly more basic childcare (i.e., ensuring that children are properly dressed, bathed, and fed), childcare management, and organized activities outside the home (Cano et al., 2019). Also, non-resident fathers spend less time with their children, leaving mothers in single-mother families with more responsibilities (McLanahan and Percheski, 2008). The involvement of fathers in childcare, on the other hand, improves family life, which in turn has downstream positive effects on children (Lamb, 2010). Increasing paternal involvement shifts some of the childcare burden away from mothers, freeing up time for mothers to work or engage in leisure activities (Kalil and Rege, 2015). Finally, maternal and paternal involvement in particular results in better resources for children. For instance, in Chile, Bares et al. (2011) studied a sample of adolescents and found that adolescents who reported more parental monitoring and more positive relationships with both parents, had lower levels of rule-breaking behavior. However, as adolescents got older, the magnitude of the association of parental monitoring and rule-breaking behavior decreased.

Children and adolescents benefit from the involvement of mothers and fathers in multiple ways. To the extent that mothers and fathers differ in their behavior and personalities, greater paternal involvement may result in greater heterogeneity in the stimuli to which children are exposed (Heckman, 2006; Hrdy, 2009; Lamb, 2010), fostering academic achievement, reducing rule-breaking behavior, and increasing interpersonal and institutional trust (Rotenberg, 1995; Ingoldsby et al., 2003; McLanahan et al., 2013; Cano et al., 2019; Chetty et al., 2020). The fathers' language skills are more predictive of children's vocabulary than the mother's and are more related with their abilities in talking to strangers (Pancsofar and Vernon-Feagans, 2006). Children and youth who share ritualistic meals and conversations with their parents score better on a range of well-being indicators (Musick and Meier, 2012). Thus, parental involvement contributes greatly to the life satisfaction of adolescents. A series of studies have shown that the subjective well-being of adolescents, in general, and life satisfaction in particular, are affected by the family context and the degree of engagement they have with their parents: a better relationship with parents improves children's satisfaction with their lives (Rask et al., 2003; Flouri and Buchanan, 2004; Levin and Currie, 2010). The effect of parental involvement on the well-being of children and adolescents is so important that it can attenuate the influence of social class and material resources (Cho, 2018). Hence, while the negative impact of deprivation on the well-being of children can deteriorate when family relationships are bad, good family relationships may avert further deterioration in subjective well-being. The direct relationship between parental involvement and the life satisfaction of early adolescents seems to be independent of their cultural background. Indeed, cross-cultural research has established the positive relationship between, for instance, parental admiration and the life satisfaction of adolescents (Schwarz et al., 2012). Based on these findings, we can state our first prediction:

**Hypothesis 1:** The evaluations of maternal and paternal involvement by early adolescents are positively related to their self-reported life satisfaction, above and beyond other important sociodemographic factors.

The evaluations of maternal and paternal involvement by adolescents may impact the life satisfaction of sons or daughters differently. The way in which mothering and fathering interact with the gender of the child could affect the perceptions of parental involvement by adolescents, resulting in differential effects on the life satisfaction of sons and daughters. Extensive literature in developmental psychology (Russell and Saebel, 1997; Leaper et al., 1998; Hastings et al., 2007) and sociology (Raley and Bianchi, 2006) has focused on whether mothers and fathers interact similarly with their sons and daughters, even when they believe that children should be treated the same, regardless of gender. Due to maturational differences, sons may elicit less verbal interaction than daughters, a gender difference in parent-child relations that can be determined very early in life and intensifies across childhood (Leaper et al., 1998). Gender composition in parent-child bonds may also elicit practices based on gender stereotypes that are still prevalent in our societies. Cross-cultural research has found that early adolescence (which includes puberty) is a period in which boys and girls are differentially socialized in gender norms and beliefs, and parents remain an important source for this process (Blum et al., 2017). Also, parents—even those who are highly educated and embrace egalitarian gender ideologies—tend to assign different chores to sons and daughters; time diary studies have documented that girls do more household work than boys (Gager et al., 1999, 2009). Parents may also sex-type their involvement with children because they believe fathers have a special knowledge to impart to sons, whereas mothers need to spend more time with daughters to properly role model them (Harris and Morgan, 1991). Gendered parenting (parental messages and behaviors that convey information about how girls and boys are supposed to behave) tends to be expressed in subtle ways in the intimate sphere (Mesman and Groeneveld, 2018). In addition, children and adolescents may contribute to this process by seeking out the parent they feel is most gender appropriate for the activity they want to engage in (Tucker et al., 2003). Besides, a growing body of research suggests that the child's gender influences the amount of time parents, particularly fathers, spend with their children (Mammen, 2011): although men and women spend more time in certain activities with children who share the same gender, the difference in parental investment is much starker for fathers. Finally, Negraia et al. (2020) recently found that the gender composition of children is an important factor that contributes to the emotional well-being of mothers and fathers while parenting, which might in turn affect the quality of their involvement with their sons and daughters. All these differences in parent-child behaviors, based on the gender composition of children, might elicit different evaluations of the parental involvement of their mothers and fathers by adolescents, in turn resulting in gendered effects on their life satisfaction. Since some form of interaction between the gender of parents and children is frequently found in the literature, we can state a non-directional hypothesis in the following terms:

**Hypothesis 2:** The gender of adolescents moderates the relationship between their evaluation of maternal and paternal involvement and their life satisfaction.

## Materials and Methods

### Participants

We used data from a survey carried out by the *Centre for Research in Inclusive Education* in Chile in 2017–2018, called *The National Survey on Student Trajectories and Transitions*, which was applied in Chilean schools. A probabilistic, stratified, two-stage sample (region-school) of students in schools in the regular educational system (urban zones) of the 16 regions of Chile was used for applying the survey between November 2017 and November 2018. For the application of the survey, the research team contacted school principals to explain the research objectives and procedures, and requested authorization to perform the survey in the school facilities. The research team provided those principals who authorized the study with printed informed-consent forms to be signed by the students' guardians, and a different questionnaire for guardians, to be answered by only one of them. Only the questionnaires of those guardians that signed the consent were considered, and only students who returned the signed guardian consent were able to participate in the study. Each student responded to a printed self-administered questionnaire, with an average response time of 60 min, on a day previously coordinated with the school principals, in the same facilities (usually, the classroom), with one research team member present to answer questions and give further directions, if necessary. The sampling framework used was the 2017 national school enrolment registry of the Chilean Ministry of Education. The survey included questionnaires for students (from first to tenth grade), guardians, teachers, and school principals. The students' questionnaire, which provided information regarding children's perception of school experiences, family structure, and a measure of their life satisfaction, was used for this paper. Since children and adolescents are usually not good at assessing their own socioeconomic status, we used the guardians' questionnaire, which requested information related to the socioeconomic background of the families of the adolescents. The responses from both questionnaires were merged using a unique student identifier.

We restricted our study to early adolescents between 5th and 8th grade. The final sample consisted of 497 students (60% females), aged 10–14 (M = 12.42, SD = 1.18) enrolled in 90 schools, and the response of the guardians of the sampled adolescents regarding socioeconomic status (although this questionnaire aimed at collecting data on the children's guardians, 97.1% of them were either the mother or father). Most of the students were enrolled in public-subsidized schools (60.1%), while 34.8% were in public schools, and 5.1% in private fee-paying schools, a school-type distribution in the sample that is similar to the national school enrolment (according to the Ministry of Education of Chile, in 2018, 35.3% of all students were enrolled in public schools; 55.5% in public-subsidized schools; and 9.3% in private schools). Descriptive statistics of the main variables are shown in Table 1.

**Table 1:** Descriptive statistics of the main variables.

**Measures**	**Mean**	**SD**	**Range**
Life satisfaction	0.00	1.00	(−3.87, 1.10)
Paternal involvement	0.00	1.00	(−2.05, 1.35)
Maternal involvement	0.00	1.00	(−3.80, 0.98)
Age	12.42	0.41	(10, 14)
School climate	0.00	1.00	(−3.43, 1.59)
SES	0.00	1.00	(−2.19, 1.94)
Ethnicity (Ref.: Not Amerindian)	0.21	–	(0, 1)
Gender (Ref.: Male)	0.60	–	(0, 1)
Nuclear family	0.63	–	(0, 1)
Two parent households	0.06	–	(0, 1)
Single parent (mother) family	0.24	–	(0, 1)
Single parent (father) family	0.03	–	(0, 1)
Other types of family	0.05	–	(0, 1)

### Main Measures

#### Adolescents' Life Satisfaction

The dependent variable was the self-reported life satisfaction of early adolescents, assessed by the *Brief Multidimensional Students' Life Satisfaction Scale* (BMSLSS), developed by Seligson et al. (2003, 2005) and validated for the Chilean context by Alfaro Inzunza et al. (2015). The BMSLSS evaluates six domains of satisfaction in students' lives (through six questions, one for each domain): family, friends, neighborhood, life at school, self, and living conditions in general. In the survey, adolescents were asked to indicate their degree of satisfaction with the six domains on a 10-point scale ranging from 0 (“not at all satisfied”) to 10 (“very satisfied”). Two sample items are “To what extent are you satisfied with your family life?” and “To what extent are you satisfied with your friends?” The mean of the six items was calculated and then transformed into standardized scores. This standardized index showed a reliability of α = 0.81.

#### Maternal and Paternal Involvement

The main predictor variables, maternal and paternal involvement, were estimated using a set of questions in the survey in which adolescents evaluated their relationship with their mothers and fathers in six dimensions involving shared time, communication, and participation in important activities. For instance, in the survey, adolescents were asked to indicate, on a 4-point scale, their feelings of closeness to their mothers and fathers (“How close do you feel to each of your parents”), ranging from 1 (“not at all close to her/him”) to 4 (“extremely close to her/him”). Also, adolescents were requested to judge five other dimensions of their relationship with both parents, in 3-point scales, such as the amount of time they share together (“Please think about the time you spend with each of your parents. Do you think your parents spend enough time with you?”), ranging from 1 (“I wish she/he spent more time with me”) to 3 (“she/he spends too much time”); discussing important decisions (“how often does each one of your parents discuss important decisions with you?”), ranging from 1 (“hardly ever”) to 3 (“often”); and parents' participation in important activities (“Regarding the participation of your parents in activities that are important to you, how often does your mother/father participate in them?”), ranging from 1 (“most of the times she/he does not attend”) to 3 (“most of the time she/he attends”). Since the response scales differed, we standardized these six measures and then averaged them. We obtained good performance in both measures: paternal involvement had an internal consistency of α = 0.86 and maternal involvement of α = 0.78.

#### Gender of the Adolescent

To explore the moderating effect of the gender of the adolescent in the relationship between parental involvement and the self-reported life satisfaction of adolescents, we also included this variable as a main measure in our analyses. It was recoded in dummy format, keeping male adolescents as a reference group (=0).

### Control Variables

We controlled for several measures that also have an impact on the subjective well-being and life satisfaction of children and adolescents, with data collected from the students' and parents' questionnaires. From the student questionnaire, we included in our analyses a measure of school climate, since recent research has highlighted that a positive school climate promotes the subjective well-being of children. Schools constitute a very important environment for children and adolescents (beyond the family), given the amount of time they spend in them and the peer relationships they build there (Steinmayr et al., 2018). We used the *School Climate Scale* included in the student questionnaire, a pool of 15 items in which the sampled adolescents evaluated three aspects of the climate in their schools (i.e., students' perceptions of norms, support from adults, and participation). The school climate scale was proposed and validated by López et al. (2014) for the Chilean context. Adolescents were requested to indicate their degree of agreement with each item (e.g., “I feel well in this school”; “Most of the students in this school are attentive and want to help”; “I can talk with my teacher when I need her/him”). Each item was evaluated on a 4-point scale, ranging from 1 (“strongly disagree”) to 4 (“strongly agree”). This scale showed good reliability performance, with α = 0.90. We also used the question from the students' questionnaire “Who do you live with?” to characterize the family structures of adolescents. Previous research suggests that family structure is responsible for important differences in the well-being of children and adolescents (Videon, 2002; Vanassche et al., 2013), although this effect might be declining in more recent times (Demo and Cox, 2000). All in all, it may be important to control for family structure when analyzing the life satisfaction of adolescents. We recoded the responses of adolescents to this question to form five groups of family structures: nuclear family, two parent households, single-parent female household, single-parent male household, and other family arrangements. Finally, we included adolescents' self-reported age and ethnicity (we codified ethnicity as a dummy variable, leaving students who reported that they do not self-recognize as belonging to any ethnic minority in Chile (i.e., not Amerindian) as the reference group (=0).

Previous research has also indicated that socioeconomic status (hereinafter, SES) is one of the most important environmental predictors of life satisfaction among children and adolescents (Ash and Huebner, 2001; Chen et al., 2016). Thus, in order to control for SES, we used two measures from the guardians' questionnaire: maximum educational attainment and the occupational status of the mother and father (separately) of the adolescents. The schooling variable contains nine categories: “incomplete basic education”; “complete basic education”; “incomplete high school”; “complete high school”; “technical training center”; “professional institute”; “university”; “master's degree”; and “PhD.” We codified all reported occupations (open question) using the *International Standard Classification of Occupations*, ISCO-08 (International Labour Organization, 2012). Thus, to characterize adolescents' SES, we took the maximum schooling level and occupational status of both parents, and then estimated the standardized mean of both measures.

### Analytical Strategy

To test the relative contribution of the main measures on the reports of life satisfaction of adolescents, above and beyond other important variables, we performed a hierarchical multivariate Ordinary Least Squares (OLS) regression analysis. Sociodemographic variables and the student's report of school climate were entered into the first block (Model 1). In the second block (Model 2), we entered family-related variables that included maternal and paternal involvement and family structure. In the final block (Model 3), we entered the interaction effects between the gender of the adolescents and their evaluation of maternal and paternal involvement. In the final model, the Shapiro-Wilk test rejects normality of the residuals (*z* = 6.81, *p* < 0.001). Visual inspection of the histogram of standardized residuals indicated that the data contained approximately normally distributed errors (slightly left-skewed). The Breusch-Pagan test rejects homoscedasticity of the residuals (χ^2^ (1) = 36.55, *p* < 0.001), so robust standard errors were estimated for correction of heteroscedasticity (HC3). Analysis of collinearity statistics show this assumption has been met, as VIF scores were well below 10, and tolerance scores above 0.2 (statistics = 1.84 and 0.54, respectively). All the analyses were conducted in Stata 16.

## Results

Descriptive statistics in Table 1 indicate that the distribution of maternal involvement is more left-skewed than paternal involvement, so the sampled adolescents broadly reported less maternal involvement than paternal involvement. Table 2 shows the correlations among the numeric variables. To control for a potential Type 1 Error accumulation due to multiple comparisons, we used the Bonferroni correction for all pairwise correlations. As expected, results in Table 2 suggest that life satisfaction is positively correlated with paternal involvement, maternal involvement, and school climate. The life satisfaction of adolescents is negatively correlated with age, although the effect size of this correlation is rather small. Low correlations between SES and life satisfaction suggests that they are somewhat independent of each other.

**Table 2:** Pearson correlations among the main measures.

	**1**	**2**	**3**	**4**	**5**
1. Life satisfaction	–				
2. Paternal involvement	0.311^***^	–			
3. Maternal involvement	0.327^***^	0.398^***^	–		
4. Age	−0.189^***^	−0.122^*^	−0.095	–	
5. School climate	0.452^***^	0.217^***^	0.282^***^	−0.241^***^	–
6. SES	0.028	0.116	0.074	−0.069	0.057

* *p < 0.05*,

*** *p < 0.001. Significance tests adjusted for multiple comparisons with Bonferroni correction*.

Table 3 shows the results of the hierarchical linear regressions predicting adolescents' life satisfaction (we present standardized coefficients). Model 1 included adolescents' sociodemographic variables. This model significantly predicted the life satisfaction of adolescents, *F* (5, 491) = 21.03, *p* < 0.001. The age-related coefficient was statistically significant, where older adolescents tended to report lower levels of life satisfaction than younger students (β = −0.082, *p* = 0.035). Female students also predicted a lower life satisfaction compared to male students (β = −0.083, *p* = 0.039). These two results are consistent with previous research indicating that life satisfaction decreases with age during adolescence, and that girls tend to report lower life satisfaction compared with boys during adolescence (Goldbeck et al., 2007). Ethnicity and SES were not significantly correlated with the life satisfaction of adolescents. In addition to these sociodemographic variables, Model 1 included school climate (as evaluated by the adolescents) as a predictor of their life satisfaction. As can be seen in Table 3, school climate had a positive and significant contribution to life satisfaction (β = 0.429, *p* < 0.001). Thus, our results also confirm previous research (Steinmayr et al., 2018) indicating that a positive school climate improves the subjective well-being of adolescents, above and beyond the effect of sociodemographic variables.

**Table 3:** Hierarchical regression models predicting adolescents' life satisfaction (BMSLSS).

**Measures**	**Model 1**			**Model 2**			**Model 3**		
	**β**	**SE**	***p-value***	**β**	**SE**	***p-value***	**β**	**SE**	***p-value***
		**(Robust)**			**(Robust)**			**(Robust)**	
Gender (Ref.: Male)	−0.082	(0.080)	0.035	−0.065	(0.077)	0.082	−0.068	(0.078)	0.072
Age	−0.083	(0.034)	0.039	−0.068	(0.033)	0.084	−0.072	(0.033)	0.068
Ethnicity (Ref.: Not Amerindian)	−0.022	(0.105)	0.605	−0.018	(0.101)	0.656	−0.019	(0.100)	0.635
SES	−0.005	(0.039)	0.892	−0.029	(0.038)	0.445	−0.033	(0.038)	0.382
School climate	0.429	(0.047)	0.000	0.357	(0.051)	0.000	0.358	(0.050)	0.000
Paternal involvement				0.148	(0.047)	0.002	0.177	(0.069)	0.010
Maternal involvement				0.154	(0.047)	0.001	−0.008	(0.076)	0.916
Family structure (Ref.: Nuclear)									
Two parent households				0.019	(0.203)	0.690	0.020	(0.200)	0.662
Single parent (mother)				−0.019	(0.097)	0.651	−0.006	(0.097)	0.895
Single parent (father)				−0.106	(0.274)	0.016	−0.111	(0.272)	0.011
Others				−0.001	(0.240)	0.980	−0.006	(0.243)	0.912
Gender x Paternal involvement							−0.022	(0.079)	0.725
Gender x Maternal involvement							0.173	(0.095)	0.026
Observations	497			497			497		
*R*^2^	0.219			0.292			0.302		
Adjusted *R*^2^	0.211			0.277			0.283		
Δ*R*^2^	–			0.073		0.000	0.010		0.043

*Standardized coefficients reported. Robust standard errors (HC3)*.

**Figure 1:**
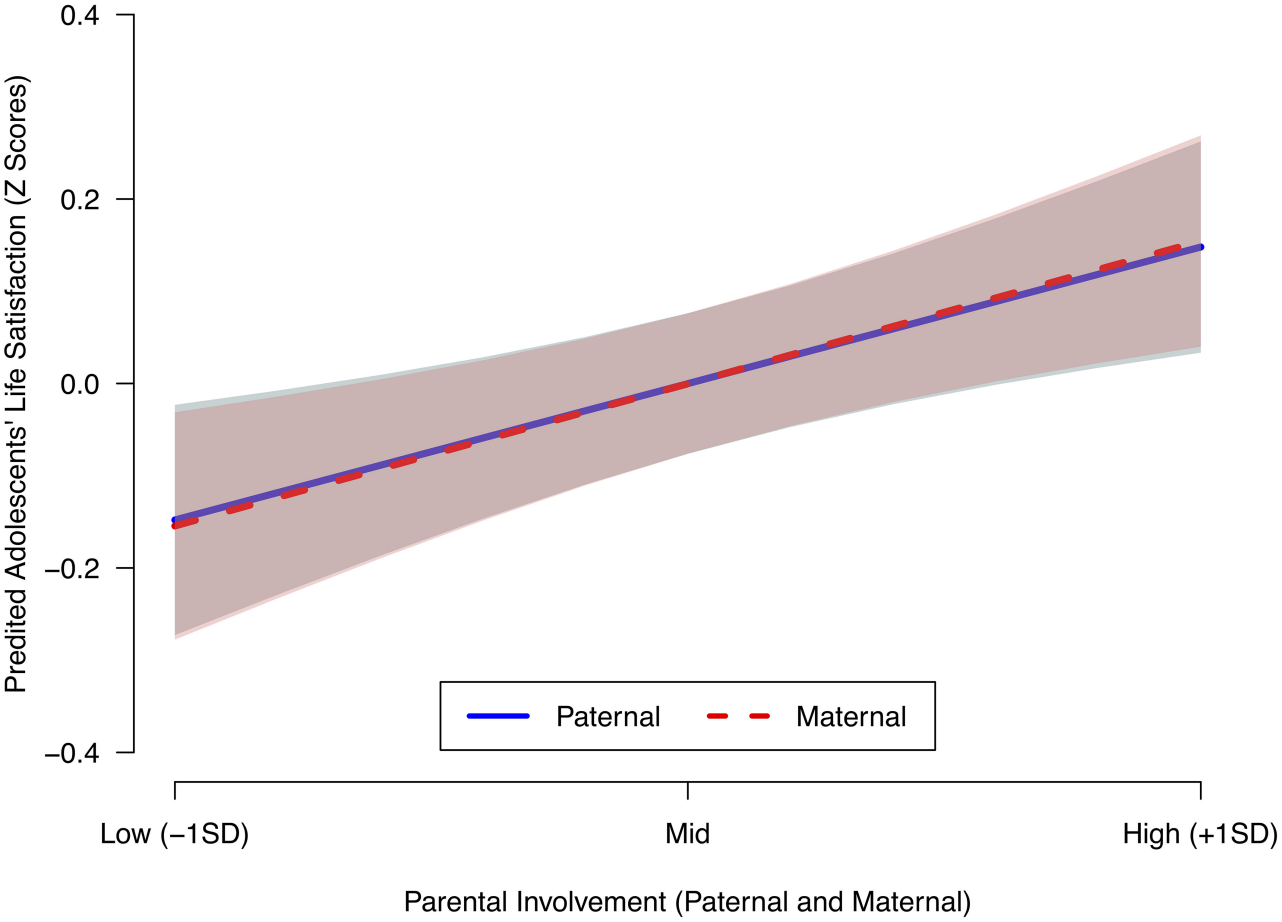
Predicted marginal effects of parental involvement on the life satisfaction of adolescents. Shaded areas represent 95% confidence intervals.

Model 2 in Table 3 included the variables related to the adolescents' families. The inclusion of these variables altered the statistical significance of gender and age, so they did not attain the conventional levels of statistical significance. Paternal involvement reported by the adolescents had a positive and statistically significant relationship with their life satisfaction (β = 0.148, *p* = 0.002). In the same way, maternal involvement had a positive and significant relationship with their life satisfaction (β = 0.154, *p* = 0.001). Figure 1 shows the predicted effects of both maternal and paternal involvement on the life satisfaction of adolescents. As can be seen, keeping all other variables constant, the effect of maternal and paternal involvement is positive and similar in magnitude. Finally, in Model 2 we see that the only family structure that revealed a statistically significant coefficient was single parent households headed by fathers, in which a lower life satisfaction of adolescents was predicted, compared to adolescents living in a nuclear family (β = 0.106, *p* = 0.016). However, we are cautious about this result, since the number of adolescents in our sample who declared that they were living in a single-parent household headed by the father is quite low (3% of the sample, as reported in Table 1). All in all, this result tends to confirm previous research suggesting that family structure tells us little about the subjective well-being of children and adolescents (Demo and Cox, 2000). Hence, the results of Model 2 confirm our first prediction (Hypothesis 1): maternal and paternal involvement are positively correlated with the self-reported life satisfaction of children during early adolescence. Overall, Model 2 was also statistically significant in predicting the dependent variable, *F* (11, 485) = 17.26, *p* < 0.001.

In the final model, we evaluated our second hypothesis: whether the gender of adolescents provides the boundary conditions for the relationship between parental involvement and their life satisfaction. Thus, in Model 3, we included two interaction effects between the involvement of parents (maternal and paternal) and the gender of adolescents in the analysis. This model also significantly predicted the outcome variable, life satisfaction, *F* (13, 483) = 14.93, *p* < 0.001. The inclusion of these terms of interaction leads to the loss of statistical significance for maternal involvement established in Model 2. Besides, the interaction of maternal involvement with the gender of the adolescent is positive and statistically significant (β = 0.173, *p* = 0.026), so the correlation of maternal involvement and life satisfaction is conditional to the gender of the adolescent. The positive relationship between paternal involvement and life satisfaction remains statistically significant, but the interaction between paternal involvement and adolescents' gender did not attain conventional levels of statistical significance (β = −0.022, *p* = 0.725). Finally, in Model 3, the simple comparison of slopes for maternal and paternal involvement indicates that the latter has a greater effect on the life satisfaction of the adolescents, suggesting that, on average, the returns of paternal involvement are greater than those of maternal involvement. Although the inclusion of the two interaction terms in Model 3 produced a statistically significant change in *R*^2^ compared to Model 2, the effect size of this change is very small (ΔR^2^ = 0.01). However, we do not believe that this small effect size plays down the importance of the uncovered moderating effect of the gender of adolescents on the relationship between maternal involvement and their life satisfaction. In the analysis of moderating effects, as Hayes (2018) has claimed, the outcome of this test is rarely interesting; “we usually are interested in the regression coefficients themselves, not the overall fit of the model” (p. 63).

**Figure 2:**
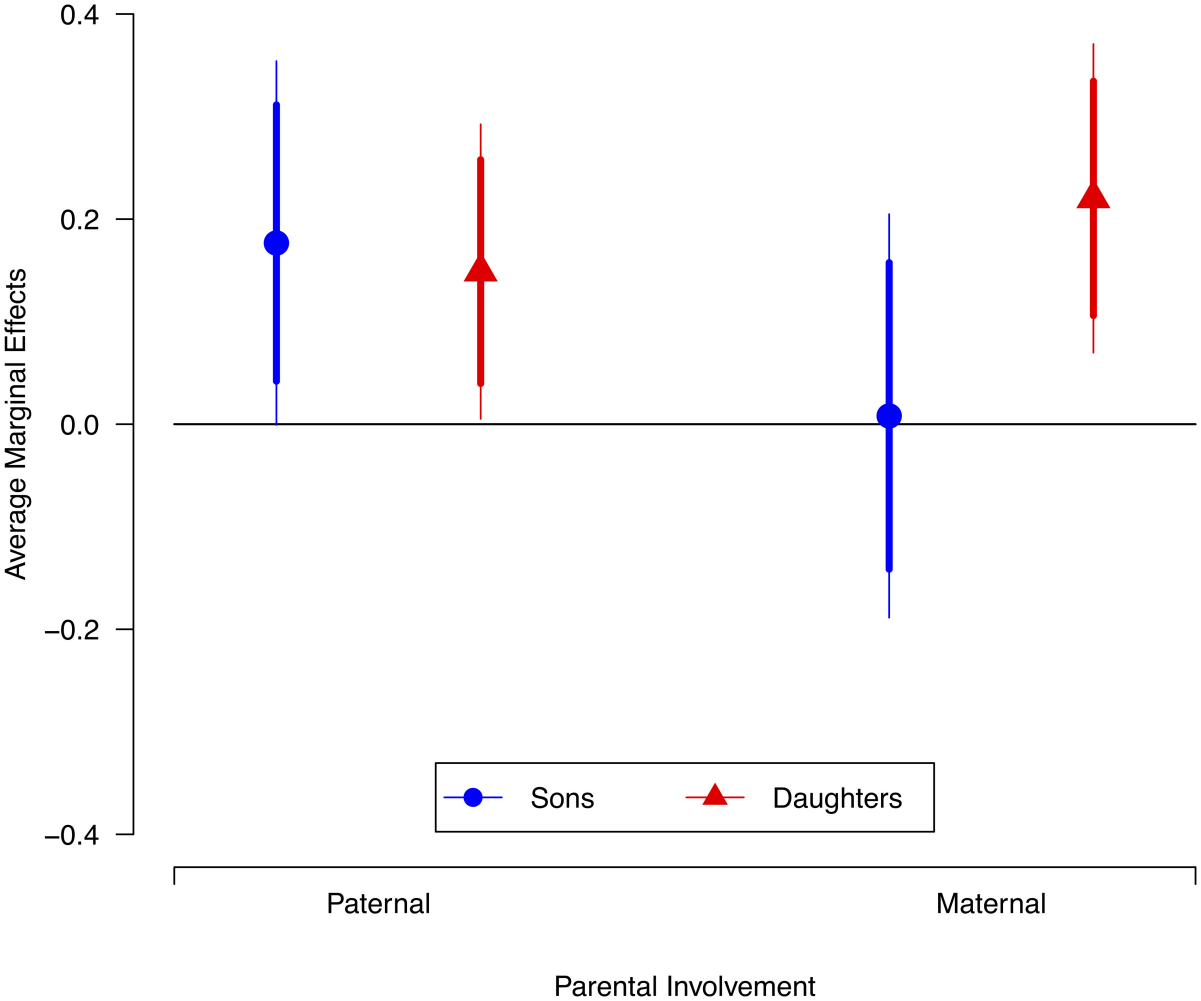
Average marginal effect of parental involvement on the life satisfaction of sons and daughters. Brackets show confidence intervals (thicker lines 95%; thinner lines 99%).

Figure 2 shows the predicted average marginal effects of these interaction effects. As can be seen, whereas paternal involvement has a positive and significant correlation with the life satisfaction of both sons and daughters (i.e., the confidence intervals do not cross the zero effect), maternal involvement only improves the life satisfaction of daughters (i.e., the confidence interval for the estimated effect of maternal involvement on sons' life satisfaction crosses the zero effect). These results confirm that the relationship of maternal involvement with the life satisfaction of the adolescents is conditional to their gender, whereas paternal involvement is not. Therefore, our second prediction is partially confirmed: the gender of the adolescent constitutes the boundary condition for the relationship between perceived maternal involvement (although not perceived paternal involvement) and their self-reported life satisfaction.

Robustness checks were conducted. In order to test a moderating effect of the SES on the aforementioned relationships between parental involvement and adolescents' gender, triple interaction terms between paternal and maternal involvement, gender, and SES were included in an additional regression model. The statistical significance of the variables included in Model 3 remained the same, including the moderation effect between maternal involvement and female adolescents. The triple interaction terms were not statistically significant. We also explored potential non-linearities in the relationship between parental involvement and the life satisfaction of the sampled adolescents, so we fitted an additional model including the quadratic term for both maternal and paternal involvement. These quadratic terms did not attain conventional levels of statistical significance and did not change the main results reported in Table 3.

## Discussion and Concluding Remarks

Early adolescence (10–14) is a transition period in which enormous physical, cognitive, emotional, and social changes occur. Although children expand their social world and gain autonomy or separateness from their parents during this developmental stage, they still need a close and supportive relationship with them. In this paper, we analyzed the relationship between early adolescents' evaluations of parental involvement and their life satisfaction, as captured by the *Brief Multidimensional Students' Life Satisfaction Scale* (BMSLSS, Seligson et al., 2003). Using a unique survey that asked early adolescents to judge parental involvement of their mothers and fathers, we were able to disentangle both effects on their self-reported life satisfaction, and we could also study the moderating effects of the gender of the adolescent on the relationship between parental involvement and life satisfaction. Results show that both paternal and maternal involvement (i.e., the extent to which adolescents feel that their mothers and fathers share enough time with them, listen to their side of the story, talk over important decisions with them) are positively correlated with the life satisfaction of adolescents. Besides, when considering gender differences, we found that the gender of the adolescents moderated the effect of maternal involvement (but not paternal involvement), so daughters (but not sons) who deemed their mothers' engagement with them to be more positive, reported greater satisfaction with their lives. More positive paternal involvement, on the other hand, correlated with higher life satisfaction for both sons and daughters. We explore some potential mechanisms explaining these correlations during early adolescence.

Firstly, in the midst of broader cultural transformations regarding the role of fathers in the intimate sphere, together with higher levels of female schooling, autonomy, and increasing participation of women in the labor market (Sayer, 2005; Gracia, 2014; Domínguez et al., 2018), our results confirm that the perceived involvement of fathers positively affects the life satisfaction of their sons and daughters during early adolescence. Furthermore, the correlation of paternal involvement with the life satisfaction of adolescents is higher than the correlation between maternal involvement and their life satisfaction (see Model 3 in Table 3). Evidently, this does not mean that paternal involvement is more important than maternal involvement. Following Flouri and Buchanan (2004), high involvement by the father already considers a similar (and usually higher) level of involvement by the mother, because she is still investing more in basic childcare and organized activities outside the home. Our results show, indeed, that adolescents reported broadly less maternal involvement than paternal involvement (see Table 1). Therefore, it is possible that the sampled adolescents underestimated the involvement of their mothers. Fathers more often concentrate on breadwinning as their primary parenting role in Chile, and they spend time with children more intermittently and under less need-based circumstances than mothers (Herrera Oesterheld and Pavicevic, 2016; Domínguez et al., 2018). Thus, paternal involvement might be more visible for adolescents and it might impact their life satisfaction more strongly, because fathers invest their time in more “meaningful” activities for early adolescents (Gracia, 2014; Cano et al., 2019). Besides, considering gender asymmetry in cultural norms (Ishizuka, 2018), early adolescents might subject their mothers to more demanding parenting standards. These lower standards for fathers could translate into higher parenting evaluations for fathers, because their behavior is compared with that of other fathers (who on average are less involved than mothers). Although these explanations seem plausible, in this study we did not have information regarding the type of activities parents are involved in with their children, so these explanations remain speculative.

Secondly, the results presented here suggest that gender composition in parent-child bonds might also affect the life satisfaction of early adolescents. Previous research has indicated that mothers and fathers play different roles in the development of children. These differences are noticeable in early adolescence, when young people undergo physical, behavioral, and social changes, and this is further complicated by the gender of the child, with distinctions in perceived family support found in mother-son, mother-daughter, father-son, and father-daughter relationships (Russell and Saebel, 1997; Raley and Bianchi, 2006). However, previous research exploring the effects that gender differences in parent-child bonds have on the life satisfaction of adolescents is inconclusive: some studies have found that adolescent subjective well-being is similarly associated with father-adolescent and mother-adolescent relationships (Sheeber et al., 2007); whereas others have found a stronger association with relationships with mothers than with fathers, particularly among daughters (Levin and Currie, 2010). Our results tend to confirm that maternal involvement has a higher impact on the life satisfaction of early adolescent daughters, whereas higher paternal involvement positively affects the life satisfaction of both sons and daughters during early adolescence. Perhaps boys are more gender-biased than girls in the way they understand and configure their relationships with their parents, at least during late-childhood and early adolescence. Perhaps involvement in shared experiences and activities in mother-daughter relationships differ in form and function from similar involvement in mother-son relationships. The moderating effect of the gender of adolescents on the relationship between the involvement of their mothers and fathers and their life satisfaction reported here deserves further investigation.

Thirdly, our results indicate that, apart from parental involvement, school climate is a strong contributor of life satisfaction during early adolescence. Indeed, we found that, among all the variables we studied in our regression models, school climate, and parental involvement were the two variables with the highest correlations on the life satisfaction of the sampled adolescents, as assessed by the standardized regression coefficients shown in Table 3. This finding is consistent with ecological approaches to subjective well-being. During early adolescence, children's lives extend beyond the family to include peers and social activities that mostly occur in the school. The family and the school are the most important socializing spheres for children and early adolescents: they spend most of their time interacting with others in these two spaces. According to the stage-environment fit approach put forth by Eccles et al. (1993), the healthy development of children is possible only if the environment fulfills the prerequisites for healthy development. Using this framework, Lawler et al. (2017) have recently stated that the subjective well-being of children (as an indication that they are developing in a healthy way) should also be impacted by a positive school climate. Indeed, we find a positive correlation between school climate and the life satisfaction of adolescents. A positive school climate seems to shape life satisfaction in early adolescence, since life satisfaction also comprises experiences at school, where they develop their social skills, bond with others, build their aspirations, and become who they are (Lawler et al., 2017; López et al., 2017).

Finally, variables commonly associated with the life satisfaction of adolescents, such as family structure and SES, did not yield statistically significant results in our analyses. Living in a nuclear family, or single-parent or two parent households, did not correlate significantly with the dependent variable. We did find a negative impact of single-parent families headed by fathers on the life satisfaction of adolescents, but we are cautious about this result because only 3% of our sample declared that they lived in this type of family. These non-significant relationships suggest that the quality of the parent-child bond is more important than the residence of the parent: non-resident parents (particularly fathers) can be positively involved with their children. Indeed, previous research has found that family structure explains little of children's subjective well-being (Demo and Cox, 2000). In Chile, 29.6% of children and youth aged 10–14 live in single-parent households; in 91.1% of them the head of the household is the mother [Ministerio de Desarrollo Social, 2017 (Ministry of Social Development)]. Thus, strategies designed to promote the subjective well-being of early adolescents should bear in mind that maternal and paternal involvement are more important in explaining the life satisfaction of adolescents than family structure. This highlights the significance of enhancing both mother-child and father-child bonds. Also, higher SES of parents was not correlated with the life satisfaction of adolescents. As recent research has demonstrated (Cho, 2018), family relationships mediate and moderate the effect of children's material deprivation on their subjective well-being. In our study, although the triple interaction among SES, gender of adolescents and parental involvement did not reach conventional levels of statistical significance (according to the robustness analyses we carried out), affluence did not correlate with the adolescents' life satisfaction, even *before* controlling for family variables. Thus, population-based strategies to improve the life satisfaction of early adolescents through better parental involvement may be equally effective across family structures and across the status hierarchy.

All in all, our results do not lend themselves to these potential explanations. One of the most important drawbacks of this study is that the survey we analyzed did not interview both fathers and mothers in the same family, so we do not have parents' assessment of their own parental involvement. However, the point of view of adolescents regarding their parents' involvement is still very informative, because we have regressed the self-reported life satisfaction of adolescents on how the actions of fathers and mothers are *perceived* by them. Evidently, perceptions of actions are not equivalent to actions themselves, but an extensive literature on psychology suggests that perceptions of parental involvement are sometimes more important than facts, and these perceptions can have long-lasting, dramatic effects during the life course (Branje et al., 2010; Susukida et al., 2016). All in all, this is an important distinction to bear in mind when interpreting the results presented here. Besides, what precise mechanism explains the reported statistical interaction between the gender of the adolescent and maternal (but not paternal) involvement remains obscure, because the analyzed data is observational and cross-sectional in nature. Additional longitudinal and cross-cultural research is needed to test the proposed explanatory mechanisms, or alternative ones. The findings presented here are a good starting point for additional research on parental involvement and the life satisfaction of early adolescents.

## Data Availability Statement

The raw data supporting the conclusions of this article will be made available by the authors, without undue reservation.

## Ethics Statement

The studies involving human participants were reviewed and approved by el Comité de Bioética de la Universidad Andrés Bello, Chile. Written informed consent to participate in this study was provided by the participants' legal guardian/next of kin.

## Author Contributions

MS, LG, and AY conceived and designed the analysis and wrote the paper. MS and LG performed the analysis. All authors contributed to the article and approved the submitted version.

## Conflict of Interest

The authors declare that the research was conducted in the absence of any commercial or financial relationships that could be construed as a potential conflict of interest.
